# GABA-activated slow spontaneous inhibitory postsynaptic currents are decreased in dorsal hippocampal dentate gyrus granule cells in an aged mouse model of Alzheimer's disease

**DOI:** 10.1177/13872877251317608

**Published:** 2025-02-16

**Authors:** Olga Netsyk, Sergiy V Korol, Jin-Ping Li, Bryndis Birnir, Zhe Jin

**Affiliations:** 1 Department of Medical Cell Biology, Uppsala University, Uppsala, Sweden; 2 Department of Medical Biochemistry and Microbiology, Uppsala University, Uppsala, Sweden

**Keywords:** Alzheimer's disease, GABA_A_ receptor, hippocampus, insulin, slow sIPSC, tg-APPSwe

## Abstract

**Background:**

Synaptic transmission dysfunction is associated with a range of neurological disorders, including Alzheimer's disease (AD). However, the role of γ-aminobutyric acid (GABA)-mediated synaptic inhibition in AD has not been fully explored.

**Objective:**

We studied basal, GABA-activated slow spontaneous synaptic currents (sIPSCs) in dentate gyrus (DG) granule cells in the dorsal hippocampus of an AD mouse model (tg-APPSwe) and investigated insulin's modulatory effects.

**Methods:**

GABA-activated slow sIPSCs were recorded in the DG granule cells by whole-cell patch­-clamp recordings in dorsal hippocampal brain slices from 5–6 (adult) and 10–12 (aged) months old wild-type (WT) and AD mice, in the presence or absence of insulin (1 nM).

**Results:**

The median 10–90% rise time of slow sIPSCs significantly decreased with age (10–12 months vs. 5–6 months) only in AD mice. The median amplitude of the slow sIPSCs was decreased in adult and aged AD mice as compared to WT mice whereas the slow sIPSCs frequency was only reduced in the aged WT mice. The median 63% decay time and total current density of the slow IPSCs was significantly decreased in the aged AD mice as compared to both WT mice and to the adult AD mice. Insulin application exerted no effect on slow sIPSCs properties in any of the animal groups.

**Conclusions:**

The characteristics of the slow sIPSCs recorded in DG granule cells of dorsal hippocampus from WT and AD mice are altered by age- and disease-state, whereas insulin has negligible effects.

## Introduction

Gamma-aminobutyric acid (GABA) is the predominant inhibitory neurotransmitter in the brain. It binds and activates two different types of receptors- the ionotropic GABA_A_ and the G-protein coupled GABA_B_ receptors generating GABA-mediated neuronal inhibition. The phasic GABA_A_ receptors inhibition can be separated into fast and slow GABA_A_ receptor mediated synaptic currents.^1,2^ The kinetics of these currents are not only different, but they are also evoked by distinct presynaptic GABAergic interneurons.^1,3,4^ The slow, spontaneous inhibitory postsynaptic currents (sIPSCs) in the principal neurons of the hippocampus are evoked in response to release from the neurogliaform interneurons and Ivy cells that release GABA by volume transmission.^1,3^ Several roles for neurogliaform interneurons and Ivy cells have been proposed including influencing neuronal network synchrony and oscillatory activity.^1,3^

It is well-established that the dorsal hippocampus (posterior hippocampus in primates) is involved in spatial learning and memory,^5^ and a gradual decline in cognitive function is one the hallmarks of Alzheimer's disease (AD). In a preclinical AD mouse model (APP^NL-G-F^), abnormal neuronal hyperactivity in brain regions including dorsal hippocampus may contribute to impaired memory retrieval.^6^ The dentate gyrus (DG) plays a crucial role in regulating cortical inputs to the hippocampus, preventing overexcitation of the hippocampal Cornu Ammonis (CA) regions. This regulation is achieved through the relatively low excitability of DG granule cells, which is attributed to both their intrinsic properties and the local GABAergic microcircuits within the dentate gyrus.^7^ While fast and tonic GABA-activated sIPSCs in DG granule cells have been well-characterized,^7,8^ slow sIPSCs mediated by the volume transmission have received less attention.

We and others have previously shown that the fast sIPSCs in hippocampal neurons are shaped by many factors including age, metabolic hormones like insulin and the amyloid-β protein (Aβ),^4,8–10^ but less is known how these conditions affect the slow IPSCs. Here we used an AD transgenic model (Tg-APPSwe mice) to explore if the slow sIPSCs were affected by age, insulin or disease. The tg-APPSwe mouse model carries a mutant version of the amyloid precursor protein (APP) that contains the Swedish mutation.^11^ This genetic alteration leads to elevated levels of the Aβ in these mice. In the APPSwe mice, the accumulation of Aβ within neurons is observed starting at 5–6 months of age.^12^ By around 12 months of age, these mice also develop extracellular Aβ plaques, as well as increased microgliosis and astrogliosis in the hippocampus.^11,12^ That insulin regulates peripheral glucose homeostasis is well established, but insulin is also increasingly recognized as a factor required for healthy brain function.^13,14^ In the DG granule cells region within the dorsal hippocampus, the effects of insulin on the fast sIPSCs are influenced by both the age and the stage of disease progression in wild-type (WT) and APPSwe mice.^8^ Here, we extended these studies with additional analyses of the same dataset as used by Hammoud et al.^8^ and examined the slow sIPSCs in adult (5–6 months) and aged (10–12 months) WT and APPSwe mice. Our results show that the slow sIPSCs are reduced in the aged APPSwe mice but are not significantly modulated by insulin.

## Methods

### Animals

All experiments were conducted in accordance with the local ethical guidelines and protocols approved by the Uppsala animal ethical committee, Swedish law and regulations based on the Directive 2010/63/EU, C129/14, C112914/15. The present study utilized adult (5–6 months old) and aged (10–12 months old) male and female C57BL/6J mice, as well as tg-APPSwe mice (bred on a C57BL/6J background). The tg-APPSwe mice were bred at Uppsala University by crossing male heterozygous tg-APPSwe with female C57BL/6J.^8^ Age-matched male and female C57BL/6J mice were used as WT controls. The animals were maintained on a 12-h light/12-h dark cycle with *ad libitum* access to water and food. The number of animals used in experiments was minimized.

To record GABA-activated currents from dorsal hippocampal DG granule cells, the following number of hippocampal slices were use: 36 slices from 6 WT at 5–6 months of age; 41 slices from 7 tg-APPSwe at 5–6 months of age; 47 slices from 8 WT mice at 10–12 months of age; and 50 slices from 13 tg-APPSwe mice at 10–12 months of age. From each slice, activity of a single neuron was recorded.

### Genotyping

The genotyping technique was performed as described previously in details.^8^ Tg-APPSwe mice overexpress transgene with human APP (isoform 695) bearing the Swedish mutation (KM670/671NL) under the murine Thy1 promoter.^12^ Mouse tail tip samples were subjected to the “HotSHOT” rapid genomic DNA isolation method to enable detection of the gene of interest.^15^ A PCR master mix was made with JumpStart^TM^ Taq DNA Polymerase (Catalog Number D6558, Sigma-Aldrich) and primer pair (APP –TYI-1-GAATCCAAGTCGGAACTCTT; APP-SQ6rw-TGTCAGGAACGAGAAGGGCA). The PCR reaction was carried out under the following parameters: an initial heating at 94°C for 2 min, followed by 30 cycles of 94°C for 15 s, 63°C for 15 s, and 72°C for 30 s, and a final incubation at 72°C for 10 min. Electrophoresis was then performed on a 1% agarose gel and the expected PCR product size was 400 bp.

### Hippocampal brain slice

The hippocampal slices were produced in accordance with the methods previously reported with slight modifications.^16,17^ At the day of experiment, the animal was euthanized by cervical dislocation and decapitated. The brain was then rapidly removed and placed in an ice-cold NMDG-based solution containing (in mM): 93 NMDG, 2.5 KCl, 1.2 NaH_2_PO_4_, 30 NaHCO_3_, 20 HEPES, 25 D-glucose, 10 MgSO_4_, 0.5 CaCl_2_, 5 sodium ascorbate, 2 thiourea, 3 sodium pyruvate, 12 N-acetyl-L-cysteine, which was oxygenated with 95% O_2_ and 5% CO_2_. The pH of this solution was adjusted to 7.3–7.4 using HCl and the osmolarity was maintained between 300–305 mOsm. Dorsal hippocampal slices were defined in the coronal plane according to Paxinos and Franklin.^18^ Coronal hippocampal slices, 350 µm thick, were then cut using a vibrating microtome Leica VT1200 S (Leica Microsystems AB, Germany). The first 3–4 consecutive hippocampal slices form the dorsal pole were collected^8,17,19^ and submerged in the NMDG-based solution for 10 min at 32°C. Afterward, the slices were transferred to a HEPES-holding solution (20–22°C) for at least 1 h prior to use. The HEPES-holding solution contained (in mM): 92 NaCl, 2.5 KCl, 1.2 NaH_2_PO4, 30 NaHCO_3_, 20 HEPES, 25 D-glucose, 2 MgSO_4_, 2 CaCl_2_, 5 sodium ascorbate, 2 thiourea, 3 sodium pyruvate, 12 N-acetyl-L-cysteine, pH 7.3–7.4 adjusted with NaOH when oxygenated with carbogen; osmolarity 305–308 mOsm.

### Electrophysiology

Whole-cell voltage-clamp recordings were performed on DG granule cells located primarily in the infrapyramidal blade of the dorsal hippocampus. The recordings were conducted at holding potential of −60 mV using Multipatch 700B amplifier controlled by pClamp 10.5 software (Molecular Devices, USA).^20^ All recordings were carried out at room temperature (20–22°C) under continuous perfusion (1.5–2 ml/min) of ACSF containing (in mM): 119 NaCl, 2.5 KCl, 1.3 MgSO_4_, 1 NaH_2_PO_4_, 26.2 NaHCO_3_, 2.5 CaCl_2_, 11 D-glucose and 3 kynurenic acid (pH 7.3–7.4 equilibrated with 95% O_2_ and 5% CO_2_; osmolarity 300–303 mOsm adjusted with sucrose). The borosilicate glass patch pipettes had a resistance of 3.5–4.1 MΩ when filled with an intracellular solution containing (in mM): 140 CsCl, 8 NaCl, 2 EGTA, 0.2 MgCl_2_, 10 HEPES, 2 MgATP, 0.3 Na_3_GTP, 5 QX314Br, pH 7.2 adjusted with CsOH, osmolarity 285–290 mOsm. Inhibitory postsynaptic currents (sIPSCs) were recorded from the granule cells for at least 5 min after a 10-min baseline stabilization period, in the continuous presence of 3 mM kynurenic acid in the ACSF solution to block excitatory synaptic transmission. Insulin (1 nM) was either acutely applied to the slices for at least 10 min or the slices were preincubated with insulin (1 nM) at room temperature for at least 30 min prior to recording. Only neurons with a stable baseline throughout the entire recording were included into the analysis. Circulation insulin levels range between pM and low nM range,^21–23^ and insulin enters the brain from the blood involves a saturable transport system.^24^ Therefore, we used 1 nM insulin in the study to avoid unspecific activation of other receptors, e.g., insulin-like growth factor-1 receptor (IGF1R).^25^

### Data analysis

The synaptic currents were analyzed using the Mini Analysis software (Synaptosoft), with the detection threshold set at a level 5 times greater than the baseline noise and checked visually.^17^ Spontaneous IPSCs having a rise time (10–90%) >5 ms were considered as slow sIPSCs. Frequency, median amplitude (pA), median rise time (10–90%) (ms), median decay time (63%) (ms) and median charge transfer (pC) for single sIPSC were analyzed by the Mini Analysis software automatically. To estimate total slow synaptic current (sIPSC*_T_*) density for each cell, we normalized total slow synaptic current (frequency x charge transfer; pA) to cell membrane capacitance value (pF), obtained after getting into the whole-cell mode. Ordinary two-way ANOVA with Fisher's LSD multiple comparison test was used to determine statistical significance (GraphPad Prism 10 Software, USA). Any p-value lower than 0.05 was interpreted as statistical significance.

### Reagents

The chemicals for electrophysiological experiments were obtained from Sigma-Aldrich (Germany), except for insulin (human, recombinant (yeast), Cat. No. 11376497001, Roche Diagnostics GmbH, Mannheim, Germany).

## Results

### Fast and slow sIPSCs recorded in DG granule cells of the dorsal hippocampus

**Figure 1.**
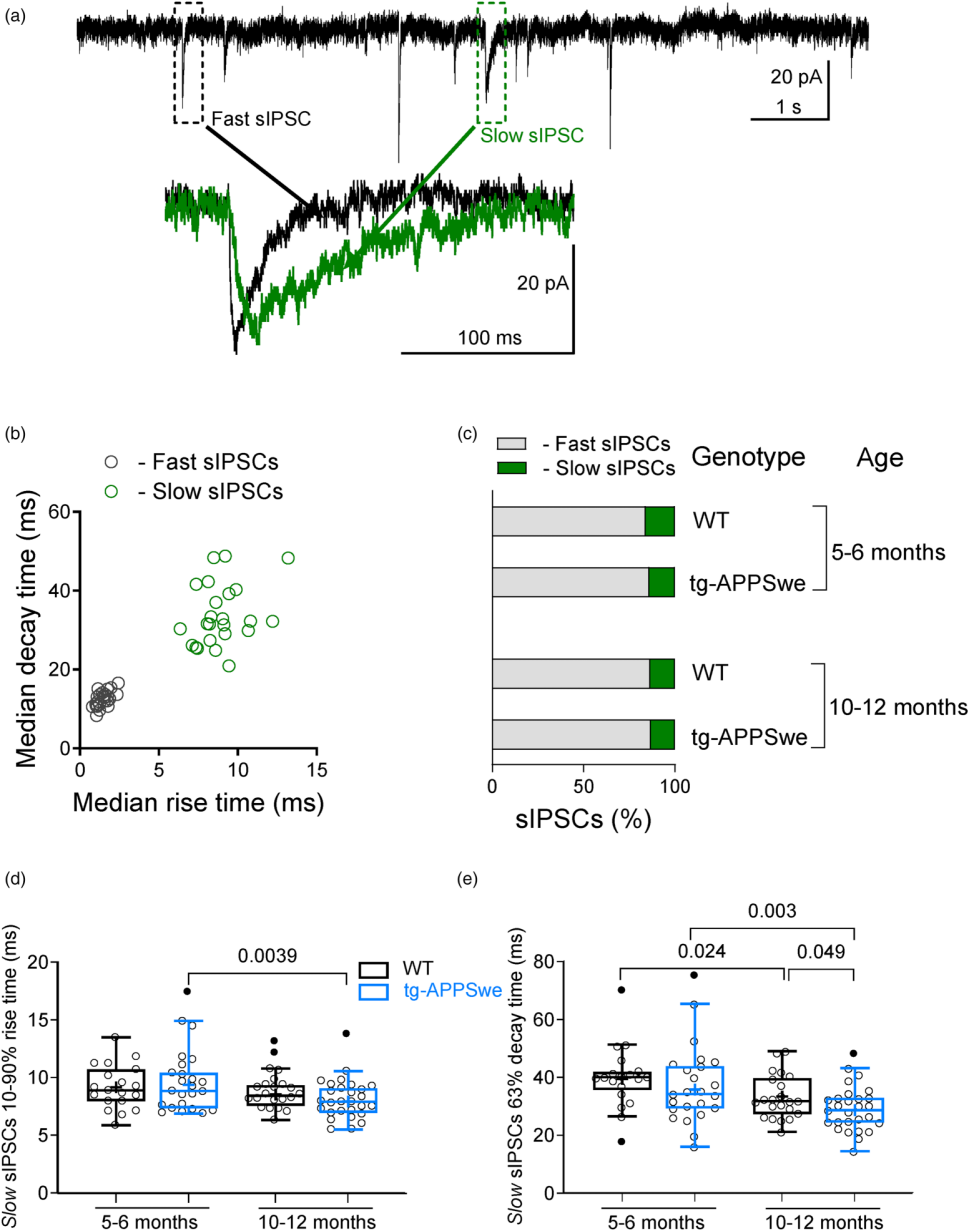
Fast and slow spontaneous IPSCs ratio and their kinetic parameters in DG granule cells of dorsal hippocampus in 5–6 or 10–12 months tg-APPSwe mice and wild-type (WT) mice. (a) Example of current recording from DG granule cells from 10–12 months old WT mice showing representative fast and slow IPSCs. (b) A graph showing the median 63% decay time plotted against the median 10–90% rise time of sIPSCs estimated for each individual DG granule cell recorded from 10–12 months old WT mice. Based on the kinetics parameters two sub-populations of sIPSCs are defined: fast with rise time ≤5 ms and decay time <20 ms *(black circles)* and slow with rise time >5 ms and decay time ≥20 ms *(green circles).* (c) A bar graph showing the percentage of the fast *(gray)* and slow *(green)* sIPSCs in DG granule cells of dorsal hippocampus in WT and tg-APPSwe mice at different age recorded in ACSF. (d, e) Kinetic parameters of slow sIPSCs. Summary plots for the median 10–90% rise time (d) and the median 63% decay time (e) of slow sIPSCs recorded from dorsal DG granule cells in hippocampal brain slices from WT and tg-APPSwe mice, age groups: 5–6 months old and 10–12 months old. Data are presented as scatter dot plot for individual values and box and whiskers plot with median value plotted as a line and the mean values shown as ‘+’. Outliers are defined by the Tukey method and marked as filled dot plot *(black circles)*. Statistical analyses are performed by excluding outliers and only statistically significant differences are marked on the graph. Ordinary two-way ANOVA with Fisher's LSD multiple comparison test was applied. V_hold _= −60 mV.

In our recordings from dorsal hippocampal DG granule cells, we identified two distinct sub-populations of GABA-activated sIPSCs (Figure 1(a)).The currents were clearly differentiated and grouped into fast and slow sIPSCs based on their kinetic properties (Figure 1(a)).The fast and slow kinetics can be distinguished by; the fast sIPSCs having rise time (10–90%) ≤5 ms and decay time (63%) <20 ms *(black circles)* and the slow sIPSCs having rise time (10–90%) >5 ms and decay time (63%) ≥20 ms *(green circles)* (Figure 1(b)). To characterize the properties of the slow sIPSCs and examine whether they are altered with age and disease progression, we studied the sIPSCs in the dorsal hippocampal DG granule cells of APPSwe mice before (5–6 months) and during (10–12 months) Aβ plaque accumulation as well as in age-matched WT mice.^4,11,12^ The majority of the sIPSCs recorded, in both 5–6 months and 10–12 months age groups in WT and APPSwe mice, were fast synaptic currents, ranging from about 84% to 87% (Figure 1(c)). Nevertheless, a significant number (13–16%) of slow events were also recorded (Figure 1(c)). In this study we focus on the slow sIPSCs, as we have previously described the characteristics of the fast sIPSCs in these mice.^8^ The median rise time (10–90%) of slow sIPSCs was markedly decreased at 10–12 months as compared to 5–6 months only in APPSwe mice (Figure 1(d)). The median decay time (63%) of the slow IPSCs was significantly reduced in APPSwe mice aged 10–12 months as compared to both WT mice and younger APPSwe mice aged 5–6 months (Figure 1(e)). Additionally, the median decay time (63%) of slow sIPSCs decreased with age (10–12 months versus 5–6 months) in WT mice (Figure 1(e)). The kinetic characteristics of the fast and slow sIPSCs are in accordance with previous reports.^2,4^

### Slow sIPSC_T_ density is reduced in aged APPSwe mice

**Figure 2.**
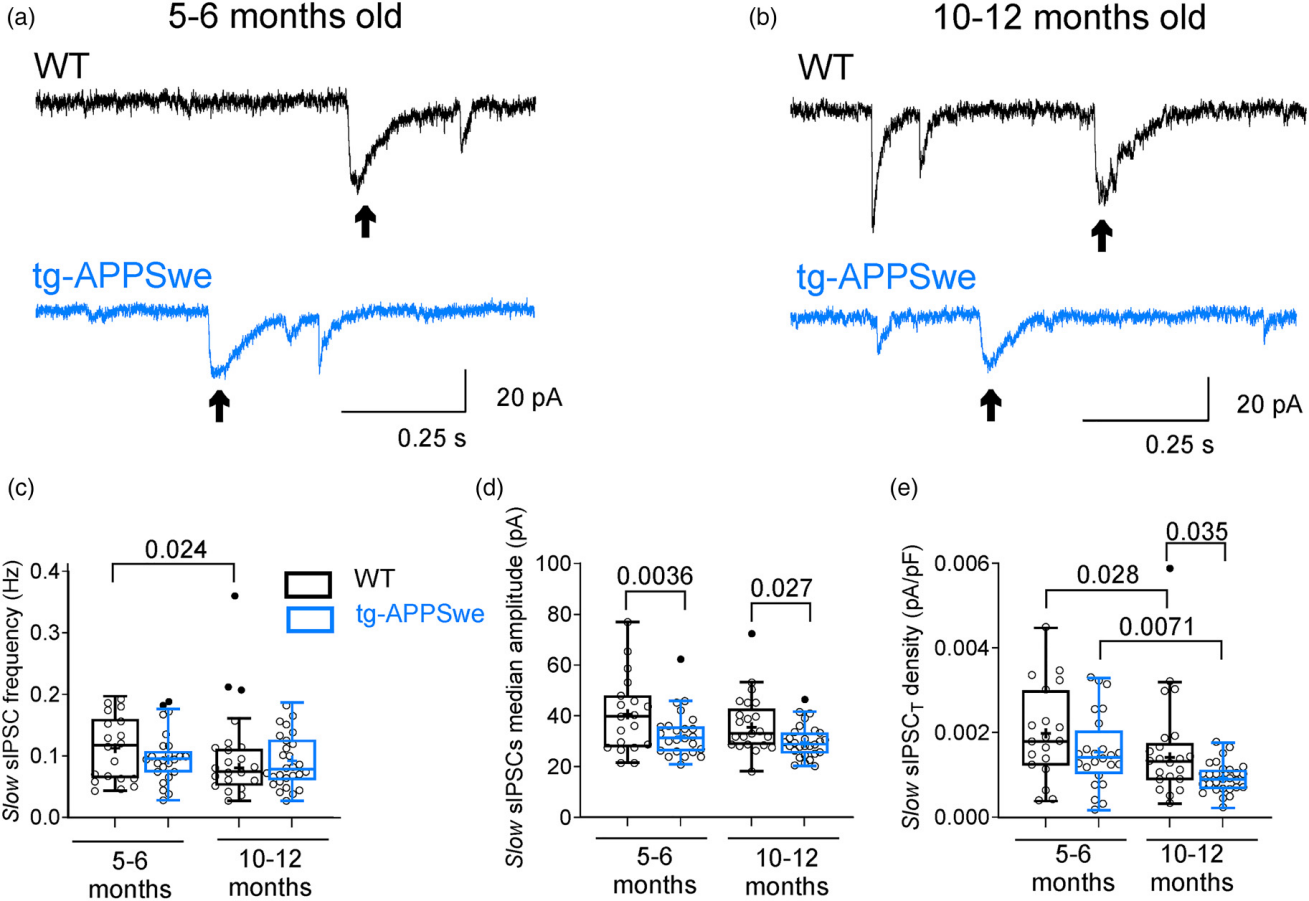
Characteristics of the slow sIPSCs in DG granule cells of the dorsal hippocampus in 5–6 and 10–12 months old tg-APPSwe mice and their WT mice. Representative voltage-clamp current traces of spontaneous IPSCs in dorsal DG granule cells from 5–6 (a) and 10–12 months old (b) WT *(black segment, upper panel)* and tg-APPSwe *(blue segment, lower panel)* mice under control conditions. Arrows indicate slow sIPSCs recorded from DG granule cells. Summary for the mean frequency (c), the median amplitude (d), and the total current density (e) of the slow sIPSCs in DG granule cells of WT and tg-APPSwe mice recorded from dorsal hippocampal slices under control conditions. Data are presented as scatter dot plot for individual cells and box and whiskers plot with median value plotted as a line and mean values shown as ‘+’. Outliers are defined by the Tukey method and marked as dot plot *(filled black circles)*. Statistical analyses are performed by excluding outliers. Ordinary two-way ANOVA with Fisher's LSD multiple comparison test was applied. All experiments were performed with parallel controls from the same animal/age group. V_hold _= −60 mV.

We further characterized properties of slow sIPSCs, including frequency, median amplitude, median charge transfer and total current density (sIPSC*_T_* density) recorded from dorsal DG granule cells in the WT and APPSwe mice aged 5–6 and 10–12 months. Examples of current traces of the slow sIPSCs are presented in Figure 2(a) and (b) for mice aged 5–6 and 10–12 months, respectively. When examined at 5–6 months of age, DG granule cells in APPSwe mice exhibited a marked reduction in the median amplitude of the slow sIPSCs compared to WT mice (Figure 2(d)). However, no notable differences were observed in the frequency, or total current density between the two groups (Figure 2(c) and (e)). In contrast, when assessed at 10–12 months of age, the median amplitude and total current density were considerably reduced in the APPSwe mice compared to the WT mice (Figure 2(d) and (e)), whereas the frequency remained similar (Figure 2(c)). Interestingly, the total current density of the slow IPSCs decreased with age (10–12 versus 5–6 months) in WT and APPSwe mice (Figure 2(e)). The frequency of the slow IPSCs was decreased with age only in WT mice (Figure 2(c)). These results indicate an age- and Aβ plaque accumulation-dependent reduction of slow IPSCs in APPSwe mice.

### Effect of insulin on slow sIPSCs in WT and APPSwe mice

**Figure 3.**
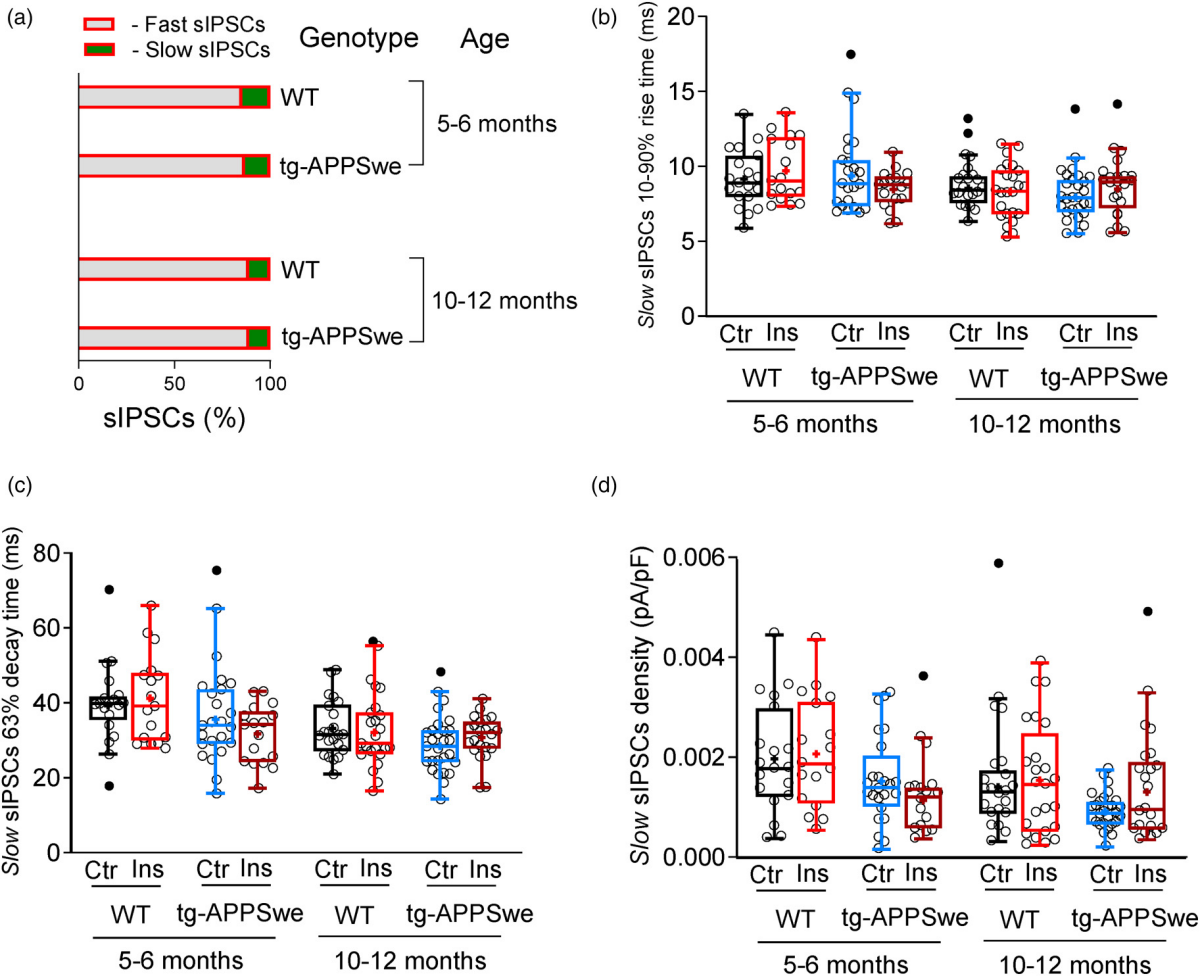
Insulin has negligible effect on the slow sIPSC*_T_* density in DG granule cells of the dorsal hippocampus in WT and tg-APPSwe mice. (a) A bar graph showing the percentage of the fast *(gray)* and slow *(green)* sIPSCs in DG granule cells of dorsal hippocampal slices treated with insulin (1 nM) in WT and tg-APPSwe mice at different age. (b, c) Kinetic parameters of slow sIPSCs. Summary plots for the median 10–90% rise time (b) and the median 63% decay time (c) of slow sIPSCs recorded from dorsal DG granule cells in hippocampal brain slices under insulin (1 nM) application from WT and tg-APPSwe mice, age groups: 5–6 months old and 10–12 months old. (d) Summary statistics for the total current (sIPSC*_T_*) density of slow sIPSCs in dorsal DG granule cells of WT and tg-APPSwe mice 5–6 and 10–12 months old recorded from hippocampal slices after pre-incubation with 1 nM insulin (Ins, *red*). All experiments were performed with parallel controls from the same animal/age group. Data are presented as scatter dot plot for individual cells and box and whiskers plot with median values plotted as a line and mean values shown as ‘+’. Outliers are defined by the Tukey method and marked as dot plot *(filled black circles)*. Statistical analyses are performed by excluding outliers. Ordinary two-way ANOVA with Fisher's LSD multiple comparison test was applied to examine the insulin effect. V_hold _= −60 mV.

We have previously shown that insulin may modulate GABA-mediated fast sIPSCs and tonic currents in APPSwe mice aged 10–12 months.^8^ Therefore, we examined whether insulin affected the slow sIPSCs properties recorded in granule cells of DG from different animal groups. Insulin did not alter the percentage of fast and slow sIPSCs (Figure 3(a)) or significantly affect the median rise time (10–90%) (Figure 3(b)), median decay time (63%) (Figure 3(c)) or total current density of the slow sIPSCs (Figure 3(d)). Additionally, insulin had no notable effect on frequency, median amplitude, or charge transfer (Table 1) in the WT and APPSwe mice aged 5–6 and 10–12 months.

**Table 1.** Insulin does not have effect on GABA-mediated slow sIPSC parameters in the dorsal DG granule cells.

	5–6 months				10–12 months			
	WT		tg-APPSwe		WT		tg-APPSwe	
	Ctr	Ins	Ctr	Ins	Ctr	Ins	Ctr	Ins
Frequency (Hz)	0.110 ± 0.012	0.109 ± 0.009	0.092 ± 0.008	0.106 ± 0.016	0.079 ± 0.008	0.090 ± 0.011	0.090 ± 0.008	0.101 ± 0.008
Amplitude (pA)	40.4 ± 3.5	36.7 ± 1.8	31.7 ± 1.4	29.5 ± 1.7	35.4 ± 1.9	34.6 ± 2.3	29.3 ± 1.1	28.3 ± 1.6
Charge transfer (pC)	1.36 ± 0.14	1.39 ± 0.15	1.08 ± 0.11	0.93 ± 0.09	1.13 ± 0.11	1.10 ± 0.11	0.86 ± 0.06	0.93 ± 0.10

Ordinary two-way ANOVA with Fisher's LSD multiple comparison test was used to compare insulin-treated cells with control cells in each animal group. The number of recorded cells ranges between 17 and 28 in each group. No statistical significance (significant level at 0.05) was detected in all comparisons. Data are presented as mean ± SEM.

## Discussion

Here we studied in DG granule cells the GABA-activated slow sIPSCs in WT and tg-APPSwe mice. The results clearly show that properties of the slow sIPSCs have already started to change in APPSwe mice aged 5–6 months and are markedly manifested in the older, 10–12 months old APPSwe mice. The findings align with decreased synaptic strength or loss of synapses in the aged APPSwe mice in comparison with WT mice.^26,27^ However, the slow sIPSCs are not noticeably affected by insulin, which contrasts with the modulation of the fast sIPSCs and tonic currents that was clearly manifested in the APPSwe mice aged 10–12 months.^8^

The slow sIPSCs are elicited by neurogliaform interneurons, which are a subtype of slow-spiking GABAergic interneurons that signal through a slow form of volume transmission that often lacks distinct postsynaptic anatomical specialisations.^1,3,4^ Selective change in the sIPSCs emerged in the APPSwe mice aged 5–6 months where the slow sIPSCs but not the fast sIPSCs were altered as compared to WT mice.^8^ The fast sIPSCs changed later and were altered in the APPSwe mice aged 10–12 months.^8^ That both the fast and the slow sIPSCs amplitudes and total current densities were reduced whereas the frequency was unaltered, may indicate a decline in the number of surface GABA_A_ receptors expressed in the DG granule cells in the APPSwe mice aged 10–12 months. However, the tonic extrasynaptic current was increased from the age of 5–6 months in the APPSwe mice.^8^ Together these findings suggest the reduction in the sIPSC*_T_* densities in the APPSwe mice aged 10–12 months is selective and may be related to smaller synapses or fewer GABA_A_ receptors located at the postsynaptic sites reached by the neurogliaform interneurons-GABA signaling. In addition, GABA spillover from parvalbumin-expressing interneurons can also evoke slow sIPSCs in newborn DG neurons.^4^ A deficit of hippocampal DG neurogenesis in APPSwe mice^28,29^ potentially contributes to the reduction of slow sIPSCs.

The observed net increase in neuronal excitatory-to-inhibitory ratio in mouse AD models is believed to be linked to the association of Aβ with synapses, which in turn leads to impaired synaptic function and disrupted network activity.^9,30,31^ Loss of inhibitory synapse has also been reported based on postmortem brain samples from AD patients.^26^ Our observations of decreased slow sIPSC*_T_* density in the 10–12 months APPSwe mice in comparison with WT mice, further supports the finding of attenuated inhibitory synaptic tone in APPSwe mice.

The influence of insulin signaling in neurons and its impact on GABA-activated currents has been studied in neurons in rat and mouse hippocampus,^8,17,32–34^ rat prefrontal cortex,^35,36^ insular cortex^37^ and mouse cerebellar granule cells.^38^ In general, insulin strengthens the GABA signaling but the level is dependent on age, cell-type, GABA_A_ receptor composition and distribution and tissue location. We have previously shown that insulin increases the fast sIPSCs current density but reduces tonic currents in hippocampal DG granule cells of APPSwe mice aged 10–12 months.^8^ At the time when plaques have formed, insulin appears to support normal inhibitory synaptic transmission in the APPSwe mice. Interestingly, the effect of insulin on the slow sIPSC*_T_* density was negligible in the APPSwe mice.

### Conclusions

In a transgenic mouse model of AD harboring the Swedish mutation (tg-APPSwe), a decrease in the GABA-evoked slow synaptic current density develops in the DG granule cells of dorsal hippocampus at 10–12 months of age. Insulin has no effects on the slow sIPSCs in contrast to the fast sIPSCs and tonic extrasynaptic currents in these neurons. This difference suggests that insulin selectively regulates the different types of GABA-activated currents.

## References

[1] Capogna M Pearce RA . GABA A, slow: causes and consequences. Trends Neurosci 2011; 34: 101–112. 21145601 10.1016/j.tins.2010.10.005

[2] Pearce RA . Physiological evidence for two distinct GABAA responses in rat hippocampus. Neuron 1993; 10: 189–200. 8382497 10.1016/0896-6273(93)90310-n

[3] Armstrong C Krook-Magnuson E Soltesz I . Neurogliaform and ivy cells: a major family of nNOS expressing GABAergic neurons. Front Neural Circuits 2012; 6: 23. 22623913 10.3389/fncir.2012.00023 PMC3353154

[4] Vaden RJ Gonzalez JC Tsai MC , et al. Parvalbumin interneurons provide spillover to newborn and mature dentate granule cells. Elife 2020; 9: e54125. 10.7554/eLife.54125 PMC7326496 32602839

[5] Strange BA Witter MP Lein ES , et al. Functional organization of the hippocampal longitudinal axis. Nat Rev Neurosci 2014; 15: 655–669. 25234264 10.1038/nrn3785

[6] Tan S Tong WH Vyas A . Impaired episodic-like memory in a mouse model of Alzheimer's disease is associated with hyperactivity in prefrontal-hippocampal regions. Dis Model Mech 2023; 16: dmm049945. 10.1242/dmm.049945 PMC10040242 36897115

[7] Coulter DA Carlson GC . Functional regulation of the dentate gyrus by GABA-mediated inhibition. Prog Brain Res 2007; 163: 235–243. 17765722 10.1016/S0079-6123(07)63014-3

[8] Hammoud H Netsyk O Tafreshiha AS , et al. Insulin differentially modulates GABA signalling in hippocampal neurons and, in an age-dependent manner, normalizes GABA-activated currents in the tg-APPSwe mouse model of Alzheimer's disease. Acta Physiol (Oxf) 2021; 232: e13623. 10.1111/apha.13623 33559388

[9] Wang Z Jackson RJ Hong W , et al. Human brain-derived Abeta oligomers bind to synapses and disrupt synaptic activity in a manner that requires APP. J Neurosci 2017; 37: 11947–11966. 29101243 10.1523/JNEUROSCI.2009-17.2017 PMC5719975

[10] Hernandez-Frausto M Bilash OM Masurkar AV , et al. Local and long-range GABAergic circuits in hippocampal area CA1 and their link to Alzheimer's disease. Front Neural Circuits 2023; 17: 1223891. 37841892 10.3389/fncir.2023.1223891 PMC10570439

[11] Philipson O Hammarstrom P Nilsson KP , et al. A highly insoluble state of Abeta similar to that of Alzheimer's disease brain is found in Arctic APP transgenic mice. Neurobiol Aging 2009; 30: 1393–1405. 18192084 10.1016/j.neurobiolaging.2007.11.022

[12] Lord A Kalimo H Eckman C , et al. The Arctic Alzheimer mutation facilitates early intraneuronal Abeta aggregation and senile plaque formation in transgenic mice. Neurobiol Aging 2006; 27: 67–77. 16298242 10.1016/j.neurobiolaging.2004.12.007

[13] Ferrario CR Reagan LP . Insulin-mediated synaptic plasticity in the CNS: anatomical, functional and temporal contexts. Neuropharmacology 2018; 136: 182–191. 29217283 10.1016/j.neuropharm.2017.12.001 PMC5988909

[14] Rolandsson O Tornevi A Steneberg P , et al. Acute hyperglycemia induced by hyperglycemic clamp affects plasma amyloid-beta in type 2 diabetes. J Alzheimers Dis 2024; 99: 1033–1046. 38728183 10.3233/JAD-230628

[15] Truett GE Heeger P Mynatt RL , et al. Preparation of PCR-quality mouse genomic DNA with hot sodium hydroxide and tris (HotSHOT). Biotechniques 2000; 29: 52, 54. 10907076 10.2144/00291bm09

[16] Ting JT Daigle TL Chen Q , et al. Acute brain slice methods for adult and aging animals: application of targeted patch clamp analysis and optogenetics. Methods Mol Biol 2014; 1183: 221–242. 25023312 10.1007/978-1-4939-1096-0_14 PMC4219416

[17] Netsyk O Hammoud H Korol SV , et al. Tonic GABA-activated synaptic and extrasynaptic currents in dentate gyrus granule cells and CA3 pyramidal neurons along the mouse hippocampal dorsoventral axis. Hippocampus 2020; 30: 1146–1157. 32533811 10.1002/hipo.23245

[18] Paxinos G Franklin KBJ . The mouse brain in stereotaxic coordinates. Compact 2nd ed. Amsterdam; Boston: Elsevier Academic Press, 2004.

[19] Milior G Di Castro MA Sciarria LP , et al. Electrophysiological properties of CA1 pyramidal neurons along the longitudinal axis of the mouse hippocampus. Sci Rep 2016; 6: 38242. 27922053 10.1038/srep38242 PMC5138623

[20] Jin Z Jin Y Birnir B . GABA-activated single-channel and tonic currents in rat brain slices. J Vis Exp 2011; 53: 2858. 10.3791/2858 PMC3196182 21788935

[21] Parks BW Sallam T Mehrabian M , et al. Genetic architecture of insulin resistance in the mouse. Cell Metab 2015; 21: 334–347. 25651185 10.1016/j.cmet.2015.01.002 PMC4349439

[22] Rijkelijkhuizen JM McQuarrie K Girman CJ , et al. Effects of meal size and composition on incretin, alpha-cell, and beta-cell responses. Metabolism 2010; 59: 502–511. 19846181 10.1016/j.metabol.2009.07.039

[23] Porksen N . The in vivo regulation of pulsatile insulin secretion. Diabetologia 2002; 45: 3–20. 11845219 10.1007/s125-002-8240-x

[24] Baura GD Foster DM Porte D Jr , et al. Saturable transport of insulin from plasma into the central nervous system of dogs in vivo. A mechanism for regulated insulin delivery to the brain. J Clin Invest 1993; 92: 1824–1830. 8408635 10.1172/JCI116773 PMC288346

[25] Chisalita SI Nitert MD Arnqvist HJ . Characterisation of receptors for IGF-I and insulin; evidence for hybrid insulin/IGF-I receptor in human coronary artery endothelial cells. Growth Horm IGF Res 2006; 16: 258–266. 16914341 10.1016/j.ghir.2006.06.003

[26] Kurucu H Colom-Cadena M Davies C , et al. Inhibitory synapse loss and accumulation of amyloid beta in inhibitory presynaptic terminals in Alzheimer's disease. Eur J Neurol 2022; 29: 1311–1323. 34331352 10.1111/ene.15043

[27] Gabitto MI Travaglini KJ Rachleff VM , et al. Integrated multimodal cell atlas of Alzheimer's disease. Nat Neurosci 2024; 27: 2366–2383. 39402379 10.1038/s41593-024-01774-5 PMC11614693

[28] Verret L Jankowsky JL Xu GM , et al. Alzheimer's-type amyloidosis in transgenic mice impairs survival of newborn neurons derived from adult hippocampal neurogenesis. J Neurosci 2007; 27: 6771–6780. 17581964 10.1523/JNEUROSCI.5564-06.2007 PMC4439193

[29] Dong H Goico B Martin M , et al. Modulation of hippocampal cell proliferation, memory, and amyloid plaque deposition in APPsw (Tg2576) mutant mice by isolation stress. Neuroscience 2004; 127: 601–609. 15283960 10.1016/j.neuroscience.2004.05.040

[30] Busche MA Chen X Henning HA , et al. Critical role of soluble amyloid-beta for early hippocampal hyperactivity in a mouse model of Alzheimer's disease. Proc Natl Acad Sci U S A 2012; 109: 8740–8745. 22592800 10.1073/pnas.1206171109 PMC3365221

[31] Busche MA Konnerth A . Impairments of neural circuit function in Alzheimer's disease. Philos Trans R Soc Lond B Biol Sci 2016; 371: 20150429. 27377723 10.1098/rstb.2015.0429 PMC4938029

[32] Wan Q Xiong ZG Man HY , et al. Recruitment of functional GABA(A) receptors to postsynaptic domains by insulin. Nature 1997; 388: 686–690. 9262404 10.1038/41792

[33] Vetiska SM Ahmadian G Ju W , et al. GABAA receptor-associated phosphoinositide 3-kinase is required for insulin-induced recruitment of postsynaptic GABAA receptors. Neuropharmacology 2007; 52: 146–155. 16890252 10.1016/j.neuropharm.2006.06.023

[34] Jin Z Jin Y Kumar-Mendu S , et al. Insulin reduces neuronal excitability by turning on GABA(A) channels that generate tonic current. Plos One 2011; 6: e16188. 10.1371/journal.pone.0016188 PMC3021545 21264261

[35] Trujeque-Ramos S Castillo-Rolon D Galarraga E , et al. Insulin regulates GABAA receptor-mediated tonic currents in the prefrontal cortex. Front Neurosci 2018; 12: 345. 29904337 10.3389/fnins.2018.00345 PMC5990629

[36] Villalobos N Ramirez-Sanchez E Mondragon-Garcia A , et al. Insulin decreases epileptiform activity in rat layer 5/6 prefrontal cortex in vitro. Synapse 2023; 77: e22263. 10.1002/syn.22263 36732015

[37] Nakaya Y Kosukegawa S Kobayashi S , et al. Insulin potentiates inhibitory synaptic currents between fast-spiking and pyramidal neurons in the rat insular cortex. Neuropharmacology 2023; 238: 109649. 37393988 10.1016/j.neuropharm.2023.109649

[38] Accardi MV Brown PM Miraucourt LS , et al. alpha6-containing GABAA receptors are the principal mediators of inhibitory synapse strengthening by insulin in cerebellar granule cells. J Neurosci 2015; 35: 9676–9688. 26134650 10.1523/JNEUROSCI.0513-15.2015 PMC6605151

